# Fungal Diversity in Barley Under Different Storage Conditions

**DOI:** 10.3389/fmicb.2022.895975

**Published:** 2022-06-22

**Authors:** Dongmei Cao, Yuhao Lou, Xiujie Jiang, Dongjie Zhang, Junmei Liu

**Affiliations:** ^1^College of Food Science, Heilongjiang Bayi Agricultural University, Daqing, China; ^2^National Coarse Cereals Engineering Research Center, Heilongjiang Bayi Agricultural University, Daqing, China; ^3^Key Laboratory of Agro-Products Processing and Quality Safety of Heilongjiang Province, Daqing, China; ^4^Heilongjiang Engineering Research Center for Coarse Cereals Processing and Quality Safety, Daqing, China; ^5^Heilongjiang Province Cultivating Collaborative Innovation Center for the Beidahuang Modern Agricultural Industry Technology, Daqing, China; ^6^College of Food Science, Jilin Agricultural University, Daqing, China

**Keywords:** barley, high-throughput sequencing technology, fungal diversity, different storage environment, deoxynivalenol

## Abstract

The diversity of fungi in barley in simulated storage environments was analyzed. Barley was stored at different temperatures (15, 25, 35°C) and relative humidity (55, 65, 75, 85 RH) for 180 and 360 days. Alpha diversity, beta diversity, species composition, and species differences were analyzed using Illumina HiSeq technology. The fungal communities in all barley samples before and after storage belonged to 3 phyla, 18 classes, 39 orders, 71 families, 103 genera, and 152 species. The relative abundance of the dominant phylum Ascomycota was 77.98–99.19%. The relative abundance of *Basidiomycota* was 0.77–21.96%. At the genus level, the dominant genera of fungi in barley initially included *Fusarium*, *Aspergillus*, *Microdochium*, *Alternaria*, and *Epicoccum*. After 360 days of storage, the dominant genera became *Epicoccum*, *Alternaria*, *Bipolar*, *Cladosporium*, *Fusarium*, and *Aspergillus*. According to Venn diagrams and principal coordinates analysis, the fungal community diversity in barley initially was much higher than in barley stored at different temperatures and humidity. The application of PLS-DA could accurately distinguish between barley stored for 180 and 360 days. Some high-temperature and high-humidity environments accelerated storage. The dominant genera differed in different storage conditions and constantly changed with increasing storage duration. *Epicoccum* was one of the dominant genera after longer storage periods. This study provides theoretical support for optimizing safe storage conditions in barley.

## Introduction

Barley is rich in protein, minerals, vitamins, dietary fiber, and other nutrients (Rashim et al., 2018), and for these reasons it has become the fourth largest cereal crop in the world (Felšöciová et al., 2021). The main barley-producing countries are Russia, Canada, Australia, the European Union countries, and North America (Jansse et al., 2018). As it is not conducive to the formation of dough and due to its lack of taste and color, barley was historically mainly used as forage grass. With improved living standards, people began to pay more attention to the nutritional value of food. Replacing wheat flour with a certain proportion of barley flour can effectively improve nutrition (Narwal et al., 2017). Products with barley as raw materials have gradually appeared, and it is now very important to control their quality and safety. One of the many applications is the beer industry, where using barley or malt as a raw material has a long history (Windes et al., 2020; Brooke et al., 2022).

Bad practices during harvest and inadequate conditions for drying, handling, packaging, storing, and transporting may contribute to fungal growth (Rajeev et al., 2010). Fungal pollution is one of the main problems in barley storage. The fungi will grow in a suitable environment and decompose the nutrients in barley, and the resultant deterioration in quality may threaten the health of consumers (Marcus et al., 2016). While different storage conditions will play a decisive role in the growth and reproduction of the fungal genera, temperature and relative humidity (RH) are the main factors affecting the growth of fungi (Yang, 2021). Appropriate environmental conditions promote the growth and reproduction of fungi, and harmful environmental conditions will inhibit the growth of fungi and even lead to death (Gostinčar et al., 2022). The most suitable growth temperature for most fungi is 15–35°C (Camardo et al., 2018). Controlling the storage temperature and limiting the growth and reproduction of harmful microorganisms can achieve safe storage. Mycotoxin are secondary metabolites produced by fungi under suitable conditions (Adányi et al., 2018). Barley is susceptible to contamination by mycotoxins (María et al., 2012), the most common being deoxynivalenol (DON) (Karolina et al., 2022), zearalenone (Mahato et al., 2021), sterigmatocystin (Morgan et al., 1986), aflatoxin (Kaale et al., 2021), and ochratoxin (Susana et al., 2008). DON, one of the most commonly detected mycotoxins, is mainly produced by *Fusarium* metabolism. Whether it has a certain correlation with *Fusarium* deserves further study.

Currently available methods for analyzing microbial flora can be considered in three categories, namely, the culture method, traditional molecular biology methods, and high-throughput sequencing technology (Hu et al., 2021). The culture method mainly uses different growth cycles of microorganisms to separate and identify cultured microorganisms, but cultured microorganisms often account for only one-tenth of the total number (Dewhirst et al., 2010). Traditional molecular biology methods mainly include Deformation Gradient Gel Electrophoresis (Aydin et al., 2015), Random Amplification Polymorphic DNA (Kermani et al., 2016), and Restriction Fragment Length Polymorphism (Camarinha-Silva et al., 2015). Although such methods can obtain accurate microbial diversity information, they have limitations in exploring the high abundance of microflora. Compared with the above traditional techniques, high-throughput sequencing technologies with high flux, high sensitivity, and good accuracy can determine millions or even tens of millions of DNA sequences simultaneously in a short time range to cover a complex microbial community. The technology has been applied to analyze microbial communities in feces (Holzhausen et al., 2021), fermented sausage (Zhang et al., 2021a), intestinal flora (Bornbusch et al., 2021), and soil (Caillon et al., 2021), and has been applied in medical science (Li et al., 2021).

Investigations of fungal diversity during storage include analyses of maize in storage for 12 months, which found that *Aspergillus*, *Fusarium*, *Wallemia*, *Sarocladium*, and *Penicillium* were the main genera (Wang et al., 2019). The main genera found in rice samples in North China and the Yangtze River valley were *Campylobacter* and *Bacteroides* (Ban et al., 2021). The main fungal species in stored wheat grains in India were *Aspergillus flavus*, *Rhizopus oryzae*, and *Eurotium amsterdam* (Kumari et al., 2019). However, to the best of our knowledge there are few reports on the structural changes of barley microbial flora and the correlations between genus and mycotoxin under different storage conditions. Therefore, studying the structural changes of the barley fungal community under different storage conditions can identify not only the optimal growth conditions of microorganisms but also apply these to control the storage environment and ensure the safety of grain storage. This study also aims to provide a solid basis for realizing safe storage of barley, reducing grain storage loss, and improving grain storage quality.

## Materials and Methods

### Preparation of Saturated Salt Solution

Storage environment humidity was simulated manually (Jang and Lee, 2021). A 55% RH environment was prepared with saturated magnesium nitrate solution (695 g/L); a 65% RH environment was prepared with saturated sodium nitrite solution (460 g/L); a 75% RH environment was prepared with saturated sodium chloride solution (360 g/L); and an 85% RH environment was prepared with saturated potassium chloride solution (342 g/L).

### Storage of Barley

Samples (500 g) of barley (variety: CK15) were packed in 12 sterile 1 L beakers, then transferred to 2 L beakers each containing different saturated salt solutions, then placed in a constant temperature incubator at three temperatures (15, 25, 35°C). Samples were taken at 180 and 360 days for subsequent analysis. The sampling method involved multipoint sampling at the upper, middle, and lower layers of the container to obtain more accurate information on community structure. Samples were named based on temperature–RH environment–storage days, and CK15 as the initial barley name.

### Determination of the DON Content

An ELISA kit (Enzyme-linked Biotechnology Co., Shanghai, China) was stored in a cryogenic refrigerator, moved to room temperature (20–25°C), and balanced for more than 1 h. Samples of weight 2 g were placed in a 50 mL centrifuge tube, 20 mL of deionized water added for 5 min, and centrifuged at 4,000 r/min for 10 min at room temperature; 0.5 mL supernatant was removed and mixed with 0.5-mL compound solution. Then, 50 μL of the above solution was taken in each microwell of the kit; each sample was made in parallel, and 50 μL of enzyme marker and antibody working liquid were added at 25°C for 30 min. After washing, 50 μL of substrate solutions A and B were added and reacted at 25°C for 15 min. Finally, 50 μL of suspension liquid was added, the absorbance value was measured at 450 nm, and the corresponding amount of mycotoxin was calculated.

### DNA Extraction and PCR Amplification

Microbial community genomic DNA was extracted from barley samples using the E.Z.N.A.^®^ DNA Kit (Omega Bio-tek, Norcross, GA, United States) according to the instructions of the manufacturer. The DNA extract was checked on 1% agarose gel, and DNA concentration and purity were determined with NanoDrop 2000 UV-vis spectrophotometer (Thermo Fisher Scientific, Wilmington, DE, United States). The hypervariable region ITS1 of the fungal ITS rRNA gene were amplified with primer pairs ITS1F (CTTGGTCATTTAGAGGAAGTAA) and ITS2R (GCTGCGTTCTTCATCGATGC) by an ABI GeneAmp^®^ 9700 PCR thermocycler (ABI, CA, United States). The PCR amplification of ITS rRNA gene was performed as follows, namely, initial denaturation at 95°C for 3 min, followed by 27 cycles of denaturing at 95°C for 30 s, annealing at 45°C for 30 s and extension at 72°C for 30 s, and single extension at 72°C for 10 min, and end at 10°C. The PCR mixtures contain 5 × TransStart FastPfu buffer 4 μL, 2.5 mM dNTPs 2 μL, forward primer (5 μM) 0.8 μL, reverse primer (5 μM) 0.8 μL, TransStart FastPfu DNA Polymerase 0.4 μL, template DNA 10 ng, and finally ddH_2_O up to 20 μL. PCR reactions were performed in triplicate. The PCR product was extracted from 2% agarose gel and purified using the AxyPrep DNA Gel Extraction Kit (Axygen Biosciences, Union City, CA, United States) according to the instructions of the manufacturer and quantified using Quantus™ Fluorometer (Promega, United States).

### Illumina MiSeq Sequencing

Purified amplicons were pooled in equimolar and paired-end sequenced on an Illumina MiSeq PE300 platform (Illumina, San Diego, CA, United States). The data presented in the study are deposited in the NCBI Sequence Read Archive (SRA) repositor, accession number SUB11386891.

### Statistical Analysis of Biological Information

The raw data were first quality-filtered to obtain high-quality sequences. Using the UPARSE (Edgar, 2013) software, statistical analysis of biological information was carried out on a 97% similar operation classification unit (OTU). Exploitation Ribosomal Database Project (RDP) Classifier (Wang et al., 2007) species classifications were annotated for each sequence. The sequences were then flattened to standardize the data for subsequent analyses. Alpha diversity was analyzed by Mothur software using Shannon, Simpson, Sobs, Ace, Chao, and Coverage as metrics (Adler et al., 2013). Community composition (Ji et al., 2017) was investigated using Heatmap graphs (Zhou et al., 2017) and Venn diagrams (Sun et al., 2021) to analyze differences in species composition. Principal coordinates analysis (PCoA) explored beta diversity based on the Bray-Curtis distance algorithm (Mitter et al., 2017). Partial Least Squares Discriminant Analysis (PLS-DA) was used to detect noticeable differences in sample grouping (Zhao et al., 2017). The differential microbiota of barley after two-stage storage were analyzed using STAMP (Ye et al., 2017).

## Results

### Alpha Diversity Analysis in the Barley Fungal Community

**FIGURE 1:**
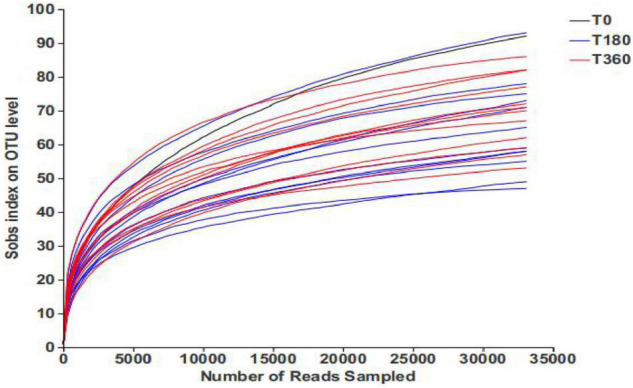
Rarefaction curves of barley samples under different storage conditions. Each curve represents a sequencing sample.

The rarefaction curves present the diversity of the samples at different sequencing numbers and also indicate whether the amount of sequencing data for the samples is reasonable. As shown in Figure 1, the rarefaction curve gradually flattened out and the sequencing data reached saturation, to cover the vast majority of species in the fungal community in barley.

The higher the Sobs, Shannon, Ace, and Chao values, the more extraordinary the community richness and diversity in the sample, while the more significant the Simpson values, the smaller the community diversity; together, these comprehensively reflect the microbial alpha diversity. Community richness reflects the number of species in the community; community diversity comprehensively reflects richness and uniformity. As shown in Table 1, all samples had coverage values above 0.9994, covering most of the sequences in the sample with an extremely low probability of being undetected. Samples stored at 35°C–75% RH (RH) for 180 days were lowest in Simpson index and highest in Shannon and Sobs indices, indicating abundant community species, but minimum values were observed in samples stored at 25°C—55/65% RH, indicating low diversity in the community. High community diversity at 360 days was observed in samples stored at 35°C–65% RH, while diversity remained low samples stored at 25°C–65% RH. At 35°C–75/65% RH, species indices were average, and these environments were suitable for most fungi, while at 25°C–65/55% RH, species compete to produce dominant genera.

**TABLE 1:** Statistical table of barley fungal community diversity indices in initial barley and after storage.

	Simpson	Shannon	Sobs	Ace	Chao	Coverage
CK15	0.154 ± 0.007	2.125 ± 0.070	101 ± 6.2	106.8 ± 9.4	105.7 ± 3.9	0.9998 ± 0.0001
a15_55_180	0.285 ± 0.009	1.675 ± 0.085	78 ± 7.2	96.9 ± 8.0	91.6 ± 4.8	0.9996 ± 0.0001
a15_65_180	0.315 ± 0.010	1.611 ± 0.082	86 ± 5.2	97.3 ± 5.8	101.1 ± 4.2	0.9997 ± 0.0001
a15_75_180	0.399 ± 0.009	1.252 ± 0.043	71 ± 5.3	100.3 ± 4.3	90.1 ± 6.9	0.9997 ± 0.0001
a15_85_180	0.351 ± 0.010	1.473 ± 0.051	80 ± 6.1	136.8 ± 6.6	140.0 ± 7.1	0.9995 ± 0.0002
a25_55_180	0.332 ± 0.010	1.597 ± 0.074	56 ± 3.0	86.3 ± 7.0	71.0 ± 6.8	0.9997 ± 0.0001
a25_65_180	0.481 ± 0.008	0.957 ± 0.089	66 ± 6.6	78.6 ± 6.5	83.5 ± 7.8	0.9997 ± 0.0002
a25_75_180	0.390 ± 0.007	1.439 ± 0.073	63 ± 5.6	100.6 ± 7.2	105.8 ± 4.8	0.9996 ± 0.0001
a25_85_180	0.340 ± 0.008	1.388 ± 0.098	65 ± 5.2	75.5 ± 6.6	76.1 ± 3.8	0.9997 ± 0.0000
a35_55_180	0.457 ± 0.014	1.161 ± 0.070	65 ± 7.9	77.1 ± 7.4	75.1 ± 3.6	0.9998 ± 0.0001
a35_65_180	0.382 ± 0.009	1.294 ± 0.082	63 ± 4.6	76.6 ± 6.3	71.7 ± 6.8	0.9998 ± 0.0001
a35_75_180	0.159 ± 0.003	2.269 ± 0.076	107 ± 8.2	115.3 ± 8.3	111.1 ± 8.9	0.9998 ± 0.0001
a35_85_180	0.239 ± 0.005	1.879 ± 0.065	85 ± 6.6	92.4 ± 6.9	89.7 ± 6.3	0.9998 ± 0.0000
a15_55_360	0.255 ± 0.007	1.731 ± 0.097	87 ± 6.1	108.84 ± 6.8	112.7 ± 6.1	0.9995 ± 0.0002
a15_65_360	0.357 ± 0.005	1.353 ± 0.058	68 ± 4.0	83.5 ± 8.6	83.1 ± 6.4	0.9996 ± 0.0001
a15_75_360	0.236 ± 0.005	1.867 ± 0.065	71 ± 7.9	79.2 ± 4.2	78.9 ± 7.7	0.9997 ± 0.0002
a15_85_360	0.330 ± 0.010	1.629 ± 0.089	90 ± 11.8	115.9 ± 6.4	115.1 ± 6.3	0.9995 ± 0.0001
a25_55_360	0.277 ± 0.003	1.690 ± 0.097	79 ± 7.6	86.9 ± 4.4	84.5 ± 6.2	0.9998 ± 0.0001
a25_65_360	0.559 ± 0.015	0.999 ± 0.086	56 ± 5.0	71.6 ± 6.4	76.0 ± 3.7	0.9996 ± 0.0001
a25_75_360	0.488 ± 0.004	1.193 ± 0.041	65 ± 4.6	84.7 ± 4.2	78.6 ± 6.4	0.9995 ± 0.0002
a25_85_360	0.316 ± 0.007	1.614 ± 0.063	72 ± 3.6	84.5 ± 4.6	81.5 ± 5.3	0.9996 ± 0.0001
a35_55_360	0.334 ± 0.005	1.612 ± 0.035	74 ± 7.8	91.7 ± 6.2	88.3 ± 5.1	0.9994 ± 0.0001
a35_65_360	0.129 ± 0.007	2.425 ± 0.081	88 ± 5.0	98.6 ± 9.4	99.7 ± 6.1	0.9996 ± 0.0002
a35_75_360	0.204 ± 0.007	1.955 ± 0.067	97 ± 9.5	113.5 ± 7.7	112.8 ± 5.4	0.9997 ± 0.0000
a35_85_360	0.307 ± 0.010	1.564 ± 0.039	56 ± 2.6	64.7 ± 7.5	62.4 ± 5.7	0.9997 ± 0.0001

*Data are expressed as the mean ± SD (n = 3).*

### Species Composition Analysis in Barley

#### Analysis of the Fungal Community Composition

**FIGURE 2:**
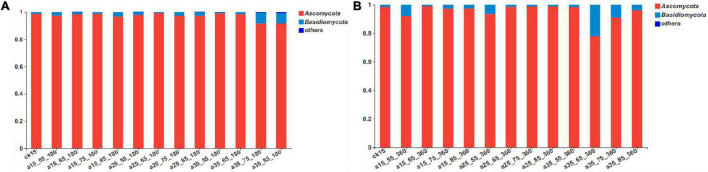
The relative abundance of fungal community phyla levels in barley after storage for 180 days **(A)** vs. 360 days **(B)**. The relative abundance of each taxon was defined as the percentage of the same taxon relative to the corresponding total sequences for each sample. The species with relative abundance ratios <1% were classified as “others.”

According to the results of the sequencing analysis, the fungal communities in the barley samples were divided into 1 domain, 1 kingdom, 3 phyla, 18 classes, 39 orders, 71 families, 103 genera, and 152 species according to the taxonomic methods. As shown in Figure 2, after 360 days of storage the fungal communities mainly contained Ascomycota and Basidiomycota. Among them, the relative abundance of Ascomycota as the dominant fungi accounted for 77.98–99.19% and Basidiomycota accounted for only 0.77–21.96%. Compared with the initial barley, at 180 days of storage the relative abundance of Basidiomycota changed greatly only in barley samples stored at 35°C–75/85% RH, increasing to 6.31–6.58% after 180 days of storage, and then to 20.73% by 360 days. During storage under the conditions 15°C–65/75% RH, 25°C–65/75/85% RH, and 35°C–55% RH, the relative abundance of Basidiomycota did not change significantly compared with the initial barley. Thus, both high temperature and long storage duration affected the growth of Basidiomycota.

**FIGURE 3:**
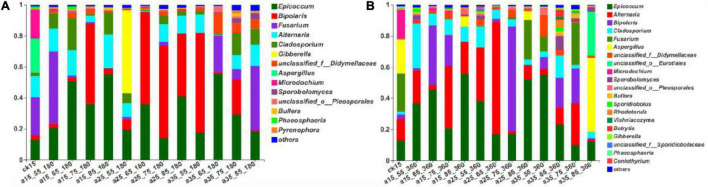
The relative abundance of fungal community genus levels in barley after storage for 180 days **(A)** vs. 360 days **(B)**. The relative abundance of each taxon was defined as the percentage of the same taxon relative to the corresponding total sequences for each sample. The species with relative abundance ratios <1% were classified as “others.”

As shown in Figure 3, the fungal community composition in the initial barley sample was relatively rich; it mainly comprised *Fusarium* (24.58%), *Aspergillus* (21.97%), *Microdochium* (18.50%), *Alternaria* (13.74%), and *Epicoccum* (13.50%). After 180 days of storage, the dominant genera changed, and the relative abundance changed to *Epicoccum*, *Bipolaris*, *Fusarium*, *Alternaria*, and *Cladosporium* (in descending order). After 360 days, there were no new dominant genera, only slight differences in relative abundance, ranging from *Epicoccum*, *Alternaria*, *Bipolaris*, *Cladosporium*, and *Fusarium* in descending order. *Epicoccum* was the dominant genus in barley storage. *Cladosporium* was present throughout the storage process and showed no significant changes in its low relative abundance. *Alternaria* showed the same trend but with high relative abundance. Storage for 180 days in the 25°C environment had similar consequences: *Bipolaris* dominated, and the relative abundance of *Fusarium* was very low. *Fusarium* was present in the initially harvested barley stored directly without treatment, resulting in similar fungal community composition in the 15°C–55% RH environment, and an obvious *Fusarium* was detected. *Gibberella* was abundant only at 25°C–55% RH–180 days. The 35°C–85% RH–360 days sample did not present the dominant genera of other samples, and mainly included *Aspergillus* and *unclassified_o_Eurotiales*. Low species diversity was observed at 25°C–65% RH, mainly composed of two genera with relative abundance of 95.34%. Species were abundant at 35°C–65/75% RH, consistent with the previously mentioned alpha diversity and indicating that multiple microorganisms began to grow in 35°C–65/75% RH. In conclusion, the dominant genera in the initial barley samples changed after storage, among which only *Epicoccum* and *Bipolaris* spores were retained. Almost all storage conditions contained *Epicoccum*, indicating that it was the dominant genus during storage.

**FIGURE 4:**
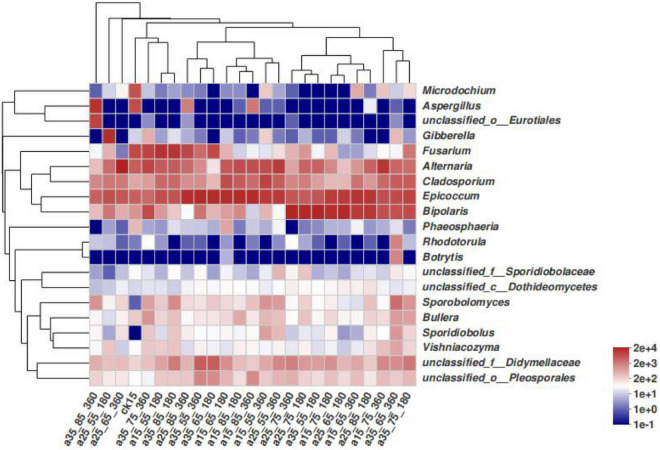
Heatmap distribution of fungal communities in barley under different storage conditions. Heatmap analysis was performed on the top 20 species of the barley samples, representing the abundance of different species in the sample by the color block color gradient.

In Figure 4, differences among species in the barley samples under different storage conditions are not noticeable. The species with high relative abundances were concentrated in a few fungi genera, and the vast majority of the storage conditions only had changes in the species abundance. The 25°C–55% RH after 180 and 360 days of storage did not cluster with other barley samples at 25°C–65% RH and 35°C–85% RH, and differed slightly in species composition, while the remaining barley could be divided into three clusters, namely, *Fusarium*, *Epicoccum*, and *Bipolaris*. It can be seen that the community structure of barley fungi changed under different conditions, and the dominant genera were significantly replaced.

#### Analysis of *Fusarium* Species in Association With DON

**FIGURE 5:**
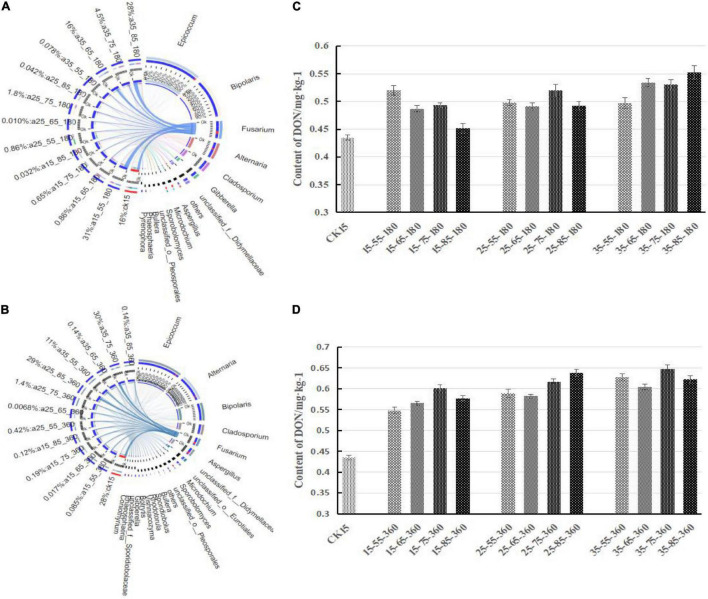
Correlation between the *Fusarium* and DON content different storage conditions. Correlations between samples and species were analyzed using Circos software to obtain the proportion of dominant species in each sample. The bar width of each genus represents the proportion of the genus in the sample [**(A)** storage 180 days; **(B)** storage 360 days]. On the right is the DON content after storage for 180 days **(C)** and 360 days **(D)**.

Figure 5 shows the correlation between *Fusarium* and DON content at 180 and 360 days, respectively. First, *Fusarium* decreased in relative abundance and was gradually replaced by other dominant genera after a long period of storage, and the overall community diversity increased with storage duration, but the overall fungal diversity of the whole storage stage was not changed. *Fusarium* species were respectively 31, 16, 4.5, and 28% in 15°C–55% RH and 35°C–65/75/85% RH at 180 days, while the DON content in the barley samples was also high. Therefore, there was a correlation between *Fusarium* species and DON, and DON content increased with the relative abundance of *Fusarium* species. The correlation still existed after 360 days of storage.

The DON content in barley showed different increasing trends under different storage conditions. The DON content was highest at 75% RH, followed by 85% RH, 65% RH, and it was lowest at 55% RH. After 360 days of storage at 15°C, the trend of increasing DON was similar in the environments of 85% RH and 65% RH, while the DON content under 85% RH at 25°C was higher than that at other temperatures. The highest DON content during the whole storage period was observed in the 35°C–75% RH environment.

#### Analysis of the Differences in Fungal Communities

**FIGURE 6:**
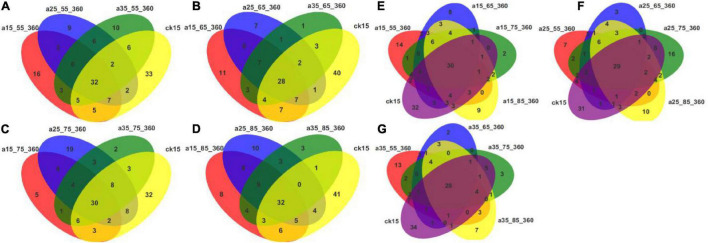
Venn diagrams of OTU distributions in barley under different storage conditions. The Venn diagrams show the common and differential OTU numbers. The different environmental conditions adopt different colors. The numbers within overlapping colors indicate the number of OTUs in common between two or more samples. The numbers within non-overlapping colors represent the number of species that differ. **(A–D)** Venn diagrams of initial barley and barley stored at 55, 65, 75, and 85% RH. **(E–G)** Venn diagrams of initial barley and barley stored at 15, 25, and 35°C.

Figure 6 shows that core and differential species existed in fungal communities of barley initially and after storage in different conditions. Considering each humidity condition separately, the numbers of differential species were greater, up to 41, in the initial barley samples compared with the samples after storage. At different temperatures, the numbers of core species were similar, while the numbers of differential species decreased. These results indicate that the fungal communities changed significantly after different storage durations in comparison to those in the initial barley samples. Multiple new species appeared, and community diversity increased significantly. Humidity conditions affected the communities more than did temperature. The samples stored at 25°C–75% RH had more differential species than samples stored in other environments, and storage at 25°C had more significant effects on fungal community variability. At 55% RH fewer differential species were observed and communities were more uniform. Fungal communities in barley stored at 35°C–55% RH differed more distinctly when compared with those in the other three humidity storage conditions. To sum up, the numbers of different species after storage indicated that storage in different environments changed the barley fungal communities.

### Comparative Analysis of the Barley Samples

#### Beta Diversity Analysis in the Barley Fungal Community

**FIGURE 7:**
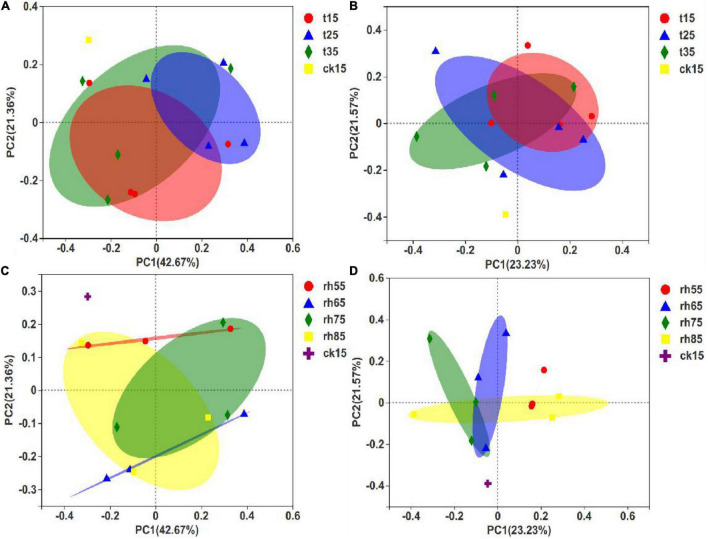
PCoA of barley fungal communities under different storage conditions. Each sample point in the figure represents a different storage condition. The closer the distance, the higher the community similarity, which shows that each storage condition has affected the initial barley and the fungal community has changed. **(A)** PCoA between different temperatures after 180 days of storage; **(B)** PCoA between different temperatures after 360 days of storage; **(C)** PCoA for differing humidity after 180 days of storage; **(D)** PCoA for differing humidity after 360 days of storage.

The PCoA of fungal communities in barley used the species abundance table to identify the potential principal components affecting differences in sample community composition by reducing dimensionality based on Euclidean distance mapping. In contrast to the results expressed in Figures 7C,D. Figures 7A,B show significant differences within groups without large differences between groups, and with an overlap between samples, indicating that RH was the main factor affecting microbial growth. Controlling the exact temperature increased the RH, and the fungal community varied greatly. In Figure 7C, 75% RH and 85% RH differed significantly, and the results by temperature showed that growth conditions in high humidity were optimized for distinctly different fungal species at each temperature. The samples stored at 55% RH had the smallest differences (Figure 7D). In low humidity, microbial growth was inhibited, and temperature had less influence. Thus, environmental humidity was the primary condition controlling the influence of temperature on the growth of microorganisms.

#### Analysis of the Differences Between the Groups

**FIGURE 8:**
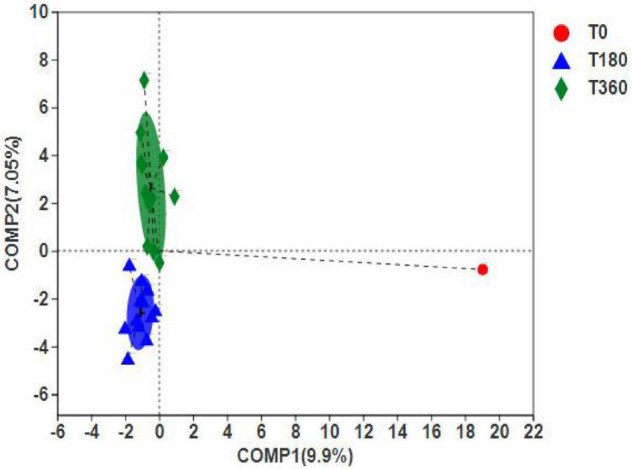
Differences between dominant genera **(A)** and significant species **(B)** in barley at different storage periods. The bar length of each genus represents the proportion of the genus in the sample (red: storage 180 days; blue: storage 360 days).

The PLS-DA can ignore random differences within groups, highlight the systematic differences between groups, and identify whether there are apparent differences in sample grouping. Figure 8 shows that the duration of storage was clustered into two distinct groups, indicating significant differences between 180 and 360 days of storage. However, the differences between initial barley and stored barley were more significant. In addition, according to the discrete sample point distribution, the flora composition at 360 days was quite different. The difference in barley stored at high temperature and high humidity for 180 days was smaller than that stored at low temperature and low humidity for 360 days. These results indicated that high-temperature and high-humidity environment could accelerate the rot of grain and reduce the storage life.

#### Significance Test of Differences Between the Two Groups

**FIGURE 9:**
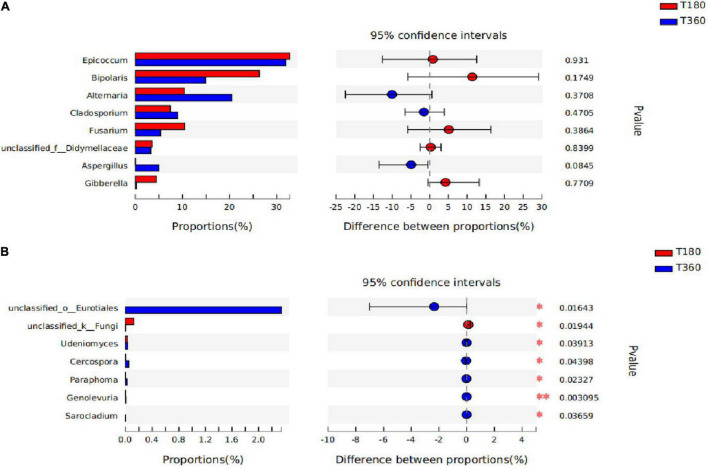
The difference between the two groups of dominant genera **(A)** and the genera are significantly different between the two groups **(B)**. The bar length of each genus represents the proportion of the genus in the sample (red: 180 days of storage; blue: 360 days of storage).

In Figure 9, the statistical significance of differences in species abundance between microbial communities in barley with different storage duration was assessed using the Wilcox rank-sum test. STAMP analysis revealed that the differential microflora at the genus level after 180 and 360 days of storage of barley, according to the figure, were not significantly different in the dominant flora, only in some differential characteristic genera (*unclassified_o_Eurotiales, unclassified_k_Fungi, Udeniomyces, Cercospora, Paraphoma, Genolevuria*, and *Sarocladium*). There was no difference between the two storage durations in the genus of *Epicoccum*, and the relative proportions of *Alternaria, Cladosporium*, and *Aspergillus* were higher in 180 d; the genera *Bipolaris, Fusarium*, and *Gibberella* had the advantage at 360 days.

## Discussion

Different genera of fungi dominated in different storage environments due to differences in requirements for growth of different fungi. The analyses of species composition in this study showed that the dominant fungi genera before and after storage included *Epicoccum*, *Alternaria*, *Bipolaris*, *Cladosporium*, *Fusarium*, and *Aspergillus*. Because the dominant genera change during the storage process, they can be classified into field fungi, storage fungi, and intermediate transition fungi, according to their respective advantages at different stages of management of contaminated crops (Francis, 2017). Field fungi can colonize the ripening grains on standing crops in the field prior to harvesting. This group includes species of the genera *Alternaria* and *Fusarium* (Lacey, 1989). *Fusarium moniliforme* was detected in a soybean study contaminated with field fungi (Liu et al., 2017). Storage fungi can be present in small numbers prior to harvesting or can contaminate grains during harvesting and increase in numbers during storage as the environmental conditions favor the growth of these fungi. Storage fungi mainly include species of the genera *Aspergillus* and *Penicillium* (Flannigan, 1978; Miller, 1995). In one study, the dominant species in stored rice mainly included *A. flavus* and *Penicillium fellutanum* (Oh et al., 2007). Transitional fungi, including *Aureobasidium*, *Geotrichum*, and *Cladosporium* are mainly affected by specific environments (Francis, 2017). Temperature, humidity, and other conditions due to geographic location can all contribute. The Venn diagrams showed that changes in temperature and humidity did not significantly affect the relevant core species, and the differences in temperature and humidity were influential in only a smaller range of species. A variety of fungal species were obtained by sequencing, but most fungi would not grow and reproduce; only a few core microorganisms were still developing (Xie et al., 2022). Under some special external environmental conditions, the dominant genus will change and have adverse effects (De Filippis et al., 2018). The PLS-DA revealed that the diversity of the fungal community in the barley was greater initially than after storage in any of the environmental conditions. It might be that the fungi in barley produced their dominant flora according to different temperatures and humidity in the early stage of storage, and those dominant flora continued to grow at the later stage of storage (Zhang et al., 2021b). Therefore, the fungal communities of the initial barley were farthest from those of the stored barley.

*Epicoccum* was the dominant genus in stored barley, but different storage conditions significantly impacted its abundance. It is a silk spore fungus found widely in ambient air, soil, and rotten vegetation (Braga et al., 2018). But *Epicoccum* is both a plant pathogen and an effective biological control agent (John et al., 2021). For example, *Epicoccum dendrobium* is effective against *Colletotrichum gloeosporioides* (Bian et al., 2021). The horizontal community composition map of genera after 180 days of storage showed that the relative abundance ratio in the 65% RH and 85% RH environments increased slightly compared with those in other humidity environments, and the growth of the coccus was also favored at 15°C. After 360 days of storage, 15°C was still the optimal growth temperature for *Epicoccum*, and the abundance was higher than at other temperatures, but the optimal humidity growth environment changed to 65% RH and 85% RH in a 25°C environment, which differed from the previous optimal condition for growth. Perhaps, such an environment is favorable for the growth of many fungal microbes, resulting in increased community diversity. Competition and specific antagonism among different species finally reduced the abundance of *Epicoccum* (Del Frari et al., 2019).

*Alternaria* was also a dominant genus in barley storage, and its abundance increased with storage duration. It is a fungal genus prevalent in the environment (Woudenberg et al., 2013), mostly pathogens that can accumulate toxic metabolites in spoiled crops (Patriarca, 2016). Evenly distributed across samples at 180 days, relative abundance was 0.84–21.70% at 180 days and 0.90–72.79% at 360 days, mainly in the 15°C –75/85% RH and 25°C –55/65% RH environments. The results of this study are somewhat different from those of previous studies (Tralamazza et al., 2016; Bretträger et al., 2022). *Alternaria* is not the most dominant strain, possibly due to differences in samples and varieties; *Cladosporium* species are widely distributed in soil and food (Tralamazza et al., 2012). *Cladosporium* was identified as a common endophytic fungus (Iturrieta-González et al., 2021), with a similar distribution pattern to *Alternaria*, and the relative abundance increased with storage duration. The low temperature of 15°C was conducive to the growth of *Cladosporium*, and its relative abundance ratio changed from 1.04–20.96% to 1.74–28.66%. Previous results also found that temperature changes may influence the colonization and growth of fungi directly through the physiology of individual organisms (Sindt et al., 2016).

*Bipolaris* was an important plant pathogen (Manamgoda et al., 2014), which mainly existed in the early storage stage. Its relative abundance in 15°C –75% RH, 35°C–55% RH, and 25°C decreased after 360 days of storage, mainly in 25°C–75% RH, up to 67.48%. Studies have shown that *Bipolaris* can maintain vitality during long-term storage. It can survive for 4–12 months without outside interference (Tanaka et al., 2001). Interestingly, the relative abundance of *Fusarium* was low in the presence of *Bipolaris*. However, 25°C was the optimal growth temperature for *Fusarium*, raising the question of whether there was a specific inhibition effect between the two. Some scholars showed that some secondary metabolites of *Bipolaris* have an antifungal effect (Afra et al., 2019). This could explain the limited growth of *Fusarium* observed in this study even when the environment was suitable. This suggests the possibility that different types of microbial metabolites inhibited growth during storage (Youenou et al., 2015).

*Fusarium* was considered to be one of the most pathogenic, phytotoxic, and mycotoxin-producing groups of microorganisms in the world (Podgórska-Kryszczuk et al., 2022). *Fusarium* causes *Fusarium* head blight (FHB) on wheat, barley, and other grains (Hao et al., 2020). During infection, *Fusarium* produces DON (Yao and Long, 2020), which contaminates grain and functions as a virulence factor to promote FHB spread throughout the wheat head (Palacios et al., 2021). The fungal community composition in the 15°C–55% RH environment was similar to that in the initial barley, and *Fusarium* was detected, perhaps because the low-temperature and low-humidity environment was not conducive to the growth and reproduction of microorganisms (De Ligne et al., 2019). The replacement of fungi genera is slow, and some field fungi do not shift to storage fungi. After 360 days of storage, the pattern of distribution of the *Fusarium* genus changed so that it mainly existed in the 25°C–85% RH and 35°C–75% RH environments. This, combined with the previously described storage distribution patterns, suggests that humidity is the main factor affecting *Fusarium* (Romero et al., 2022). The DON content was used as the index to evaluate the presence of *Fusarium*, and a positive correlation was found between the DON content and the relative abundance of *Fusarium*. It can be noted that first, with increases in storage temperature and relative environmental humidity, mycotoxin will accumulate (Patriarca, 2019). Second, harvested barley is accompanied by a fungal system in the field growing environment (Mannaa and Kim, 2017). DON is a secondary metabolite of *Fusarium*, which gradually produces metabolites after a period of storage where toxic fungi have an excellent growth opportunity, and their metabolism will increase (Xu et al., 2021).

## Conclusion

In this study, Illumina HiSeq high-throughput sequencing was used to analyze the fungal diversity in barley samples stored in 12 different storage conditions for 180 and 360 days. The fungal communities in the barley belonged to 3 phyla, 18 classes, 39 orders, 71 families, 103 genera, and 152 species. Compared with the initial barley, the community composition and the relative abundance of species changed after storage under different conditions. The initially dominant genera *Fusarium*, *Aspergillus*, and *Microdochium* were replaced by *Epicoccum*, *Alternaria*, *Bipolaris*, and *Cladosporium*.

The relative abundances in the fungal communities changed under all storage conditions, with the lowest species diversity under 25°C–65% RH, and more abundance under 35°C–65/75% RH. In contrast, species with high abundance were concentrated in a few genera, and samples in the vast majority of storage conditions only changed in species abundance. Correlation analysis showed a positive correlation between *Fusarium* and DON content. The differential and core fungal species in the initial and stored barley were illustrated using Venn diagrams. PCoA showed significant differences among fungal communities in barley under different temperature and humidity conditions before and after storage. RH has a greater impact on microorganisms than temperature. PLS-DA showed that the fungal communities of the two storage durations had distinctive classification characteristics. Some high-temperature and humidity conditions accelerated barley spoilage and fungal propagation. The species composition diagram showed that 15°C–55% RH was similar to the initial barley composition, which would be a good storage condition. Therefore, to ensure the quality of barley and the health of consumers, barley should not be stored in a high-temperature and high-humidity environment. Barley should be stored in a dry and low-temperature environment to avoid the adverse influence of high temperature and high humidity on grain. For example, in an environment of 15°C–55% RH. This study highlighted changes in fungal diversity in barley under different storage conditions. The lessons learned from this study are critical for safe storage and loss mitigation.

## Data Availability Statement

The original contributions presented in this study are included in the article/supplementary material, further inquiries can be directed to the corresponding author/s.

## Author Contributions

DZ was responsible for the design and overall management of the entire experiment. YL performed the experimental methods and collected the data. DC analyzed the data and wrote the manuscript. XJ and JL revised the improvement format. All authors have read and agreed to the published version of the manuscript.

## Conflict of Interest

The authors declare that the research was conducted in the absence of any commercial or financial relationships that could be construed as a potential conflict of interest.

## Publisher’s Note

All claims expressed in this article are solely those of the authors and do not necessarily represent those of their affiliated organizations, or those of the publisher, the editors and the reviewers. Any product that may be evaluated in this article, or claim that may be made by its manufacturer, is not guaranteed or endorsed by the publisher.

## References

[B1] Adányi N. Nagy A. G. Takács B. Szendrõ I. Szakacs G. Szûcs R. (2018). Sensitivity enhancement for mycotoxin determination by optical waveguide lightmode spectroscopy using gold nanoparticles of different size and origin. *Food Chem.* 267 10–14. 10.1016/j.foodchem.2018.04.089 29934142

[B2] Adler C. J. Dobney K. Weyrich L. S. Kaidonis J. Walker A. W. Haak W. (2013). Sequencing ancient calcified dental plaque shows changes in oral microbiota with dietary shifts of the Neolithic and Industrial revolutions[J]. *Nat. Genet.* 45:450. 10.1038/ng.2536 23416520 PMC3996550

[B3] Afra K. Rosella S. Sahar S. Dominique L. M. (2019). Diversity of natural products of the genera Curvularia and Bipolaris[J]. *Fung. Biol. Rev.* 33 101–122. 10.1016/j.fbr.2018.09.002

[B4] Aydin S. Shahi A. Ozbayram E. G. Ince B. Ince O. (2015). Use of PCR-DGGE based molecular methods to assessment of microbial diversity during anaerobic treatment of antibiotic combinations[J]. *Bioresour. Technol.* 192 735–740. 10.1016/j.biortech.2015.05.086 26101963

[B5] Ban Y. H. Li X. Li Y. Q. Li X. Y. Li X. Wang X. J. (2021). Comparative analysis of paddy straw-degrading consortia in China using high-throughput sequencing. *Appl. Soil Ecol.* 2021:167. 10.1016/J.APSOIL.2021.104077

[B6] Bian J. Y. Fang Y. L. Song Q. Sun M. L. Yang J. Y. Ju Y. W. (2021). The fungal endophyte Epicoccum dendrobii as a potential biocontrol agent against Colletotrichum gloeosporioides.[J]. *Phytopathology* 111 293–303. 10.1094/PHYTO-05-20-0170-R 32748735

[B7] Bornbusch S. L. Harris R. L. Grebe N. M. Roche K. Dimac-Stohl K. Drea C. M. (2021). Antibiotics and fecal transfaunation differentially affect microbiota recovery, associations, and antibiotic resistance in lemur guts[J]. *Anim. Microb.* 3 65–65. 10.1186/s42523-021-00126-z 34598739 PMC8485508

[B8] Braga R. M. Padilla G. Araújo W. L. (2018). The biotechnological potential of Epicoccum spp.: diversity of secondary metabolites[J]. *Crit. Rev. Microb.* 44 1–20. 10.1080/1040841x.2018.1514364 30369284

[B9] Bretträger M. Becker T. Gastl M. (2022). Screening of Mycotoxigenic Fungi in Barley and Barley Malt (Hordeum vulgare L.) Using Real-Time PCR-A Comparison between Molecular Diagnostic and Culture Technique. *Foods* 11:1149. 10.3390/foods11081149 35454736 PMC9030328

[B10] Brooke S. C. Harmonie B. Sarah W. Patricia A. Luis C. Scott F. (2022). Genetic basis of barley contributions to beer flavor. *J. Cereal Sci.* 2022:104. 10.1016/J.JCS.2022.103430

[B11] Caillon F. Besemer K. Peduzzi P. Schelker J. (2021). Soil microbial inoculation during flood events shapes headwater stream microbial communities and diversity[J]. *Microb. Ecol.* 82 591–601. 10.1007/s00248-021-01700-3 33532913 PMC8463373

[B12] Camardo L. M. Decontardi S. Battilani P. (2018). Modelling the sporulation of some fungi associated with cheese, at different temperature and water activity regimes. *Int. J. Food Microbiol.* 278 52–60. 10.1016/j.ijfoodmicro.2018.04.023 29702316

[B13] Camarinha-Silva A. Wos-Oxley M. L. Jáuregui R. Becker K. Pieper D. H. (2015). Validating T-RFLP as a sensitive and high-throughput approach to assess bacterial diversity patterns in human anterior nares[J]. *FEMS Microbiol. Ecol.* 79 98–108. 10.1111/j.1574-6941.2011.01197.x 22066869

[B14] De Filippis F. La Storia A. Villani F. Ercolini D. (2018). Strain-level diversity analysis of *Pseudomonas* fragi after in situ pangenome reconstruction shows distinctive spoilage-associated metabolic traits clearly selected by different storage conditions. *Appl. Env. Microbiol.* 2018:85. 10.1128/aem.02212-18 30366996 PMC6293103

[B15] De Ligne L. Vidal-Diez de Ulzurrun G. Baetens J. M. Van den Bulcke J. Van Acker J. De Baets B. (2019). Analysis of spatio-temporal fungal growth dynamics under different environmental conditions. *IMA Fungus* 10:7. 10.1186/s43008-019-0009-3 32647616 PMC7325663

[B16] Del Frari G. Cabral A. Nascimento T. Boavida Ferreira R. Oliveira H. (2019). Epicoccum layuense a potential biological control agent of esca-associated fungi in grapevine.[J]. *PLoS One* 14:e0213273. 10.1371/journal.pone.0213273 30913218 PMC6435229

[B17] Dewhirst F. E. Chen T. Izard J. Paster B. J. Tanner A. C. Yu W. H. (2010). The Human Oral Microbiome[J]. *J. Bacteriol.* 192 5002–5017. 10.1128/JB.00542-10 20656903 PMC2944498

[B18] Edgar R. C. (2013). UPARSE: highly accurate OTU sequences from microbial amplicon reads. *Nat. Methods* 10 996–998. 10.1038/nmeth.2604 23955772

[B19] Felšöciová S. Kowalczewski P. Ł Krajcovic T. Drab S. Kacaniova M. (2021). Effect of Long-Term Storage on Mycobiota of Barley Grain and Malt. *Plants* 10:1655. 10.3390/plants10081655 34451699 PMC8401099

[B20] Flannigan B. (1978). Primary contamination of barley and wheat grain storage fungi[J]. *Transact. Br. Mycol. Soc.* 71 37–42. 10.1016/S0007-1536(78)80005-9

[B21] Francis F. L. (2017). Integrated management of the risks of stored grain spoilage by seedborne fungi and contamination by storage mould mycotoxins – An update[J]. *J. Stor. Prod. Res.* 71 22–40. 10.1016/j.jspr.2016.10.002

[B22] Gostinčar C. Zalar P. Gunde C. N. (2022). No need for speed: slow development of fungi in extreme environments[J]. *Fung. Biol. Rev.* 39 1–14. 10.1016/J.FBR.2021.11.002

[B23] Hao G. McCormick S. Usgaard T. Tiley H. Vaughan M. M. (2020). Characterization of Three Fusarium graminearum Effectors and Their Roles During Fusarium Head Blight. *Front. Plant Sci.* 11:579553. 10.3389/fpls.2020.579553 33329641 PMC7734257

[B24] Holzhausen E. A. Nikodemova M. Deblois C. L. Barnet J. H. Peppard P. E. Suen G. (2021). Assessing the impact of storage time on the stability of stool microbiota richness, diversity, and composition[J]. *Gut Pathog.* 13 75–75. 10.1186/s13099-021-00470-0 34930464 PMC8686582

[B25] Hu T. Chitnis N. Monos D. Dinh A. (2021). Next-generation sequencing technologies: an overview[J]. *Hum. Immunol.* 82 801–811. 10.1016/j.humimm.2021.02.012 33745759

[B26] Iturrieta-González I. García D. Gené J. (2021). Novel species of Cladosporium from environmental sources in Spain. *MycoKeys* 77 1–25. 10.3897/mycokeys.77.60862 33510579 PMC7803722

[B27] Jang B. K. Lee C. H. (2021). Effect of temperature and relative humidity on the viability and longevity of eastern bracken (Pteridium aquilinum var. latiusculum) spores for long-term storage. *Scientia Horticulturae* 288:110362. 10.1016/j.scienta.2021.110362

[B28] Jansse E. M. Liu C. Fels-Klerx H. J. (2018). Fusarium infection and trichothecenes in barley and its comparison with wheat[J]. *World Mycotoxin J.* 11 33–46. 10.3920/wmj2017.2255 29510743

[B29] Ji P. Rhoads W. J. Edwards M. A. Pruden A. (2017). Impact of water heater temperature setting and water use frequency on the building plumbing microbiome[J]. *ISME J.* 11:1318. 10.1038/ismej.2017.14 28282040 PMC5437349

[B30] John D. T. Edzel E. Mark A. B. (2021). Epicoccum species: ubiquitous plant pathogens and effective biological control agents. *Eur. J. Plant Pathol.* 2021 1–13. 10.1007/S10658-021-02207-W

[B31] Kaale L. D. Kimanya M. E. Macha I. J. Mlalila N. (2021). Aflatoxin contamination and recommendations to improve its control: a review[J]. *World Mycotoxin J.* 14 27–40. 10.3920/WMJ2020.2599 29510743

[B32] Karolina T. R. Grazina J. Janić H. E. Vadims B. Iveta P. Dovile K. (2022). Challenges of Lactobacillus fermentation in combination with acoustic screening for deoxynivalenol and deoxynivalenol conjugates reduction in contaminated wheat - based products. *Food Control* 2022:134. 10.1016/J.FOODCONT.2021.108699

[B33] Kermani F. Shams-Ghahfarokhi M. Gholami-Shabani M. Razzaghi-Abyaneh M. (2016). Diversity, molecular phylogeny and fingerprint profiles of airborne Aspergillus species using random amplified polymorphic DNA[J]. *World J. Microbiol. Biotechnol.* 32:96. 10.1007/s11274-016-2052-1 27116962

[B34] Kumari R. Jayachandran L. E. Ghosh A. K. (2019). Investigation of Diversity and Dominance of Fungal Biota in Stored Wheat Grains from Governmental Warehouses in West Bengal, India. *J. Sci. Food Agricult.* 99 3490–3500. 10.1002/jsfa.9568 30623426

[B35] Lacey J. (1989). Pre- and post-harvest ecology of fungi causing spoilage of foods and other stored products. *Soc. Appl. Bacteriol. Symp. Series* 18 11S–25S. 10.1111/j.1365-2672.1989.tb03766.x 2508232

[B36] Li P. Wang K. Qiu S. Lin Y. Xie J. Li J. (2021). Rapid identification and metagenomics analysis of the adenovirus type 55 outbreak in Hubei using real-time and high-throughput sequencing platforms[J]. *Infect. Genet. Evol.* 93 104939–104939. 10.1016/j.meegid.2021.104939 34029726

[B37] Liu J. Deng J. C. Yang C. Q. Huang N. Chang X. L. Zhang J. (2017). Fungal Diversity in Field Mold-Damaged Soybean Fruits and Pathogenicity Identification Based on High-Throughput rDNA Sequencing[J]. *Front. Microb.* 8:779. 10.3389/fmicb.2017.00779 28515718 PMC5413577

[B38] Mahato D. K. Devi S. Pandhi S. Sharma B. Maurya K. K. Mishra S. (2021). Occurrence, Impact on Agriculture, Human Health, and Management Strategies of Zearalenone in Food and Feed: a Review. *Toxins* 13 92–92. 10.3390/TOXINS13020092 33530606 PMC7912641

[B39] Manamgoda D. S. Rossman A. Y. Castlebury L. A. Crous P. W. Madrid H. Chukeatirote E. (2014). The genus Bipolaris. *Stud. Mycol.* 79 221–288. 10.1016/j.simyco.2014.10.002 25492990 PMC4255534

[B40] Mannaa M. Kim K. D. (2017). Influence of Temperature and Water Activity on Deleterious Fungi and Mycotoxin Production during Grain Storage[J]. *Mycobiology* 45 240–254. 10.5941/myco.2017.45.4.240 29371792 PMC5780356

[B41] Marcus S. Stefan H. Lorenzo D. C. Martin D. Karl S. Emanuele Z. (2016). Impact of fungal contamination of wheat on grain quality criteria[J]. *J. Cereal Sci.* 69 95–103. 10.1016/j.jcs.2016.02.010

[B42] María I. V. Elena G. P. Elena L. Adela L. C. (2012). Co-occurrence of mycotoxins in Spanish barley: a statistical overview[J]. *Food Control* 28 295–298. 10.1016/j.foodcont.2012.04.046

[B43] Miller J. D. (1995). Fungi and mycotoxins in grain: implications for stored product research[J]. *J. Stored Prod. Res.* 31 1–16. 10.1016/0022-474X(94)00039-V

[B44] Mitter E. K. Freitas J. R. Germida J. J. (2017). Bacterial Root Microbiome of Plants Growing in Oil Sands Reclamation Covers[J]. *Front. Microb.* 8:849. 10.3389/fmicb.2017.00849 28559882 PMC5432656

[B45] Morgan M. R. A. Kang A. S. Chan W. S. (1986). Production of antisera against sterigmatocystin hemiacetal and its potential for use in an enzyme-linked immunosorbent assay for sterigmatocystin in barley[J]. *J. Sci. Food Agricult.* 37 873–880. 10.1002/jsfa.2740370909

[B46] Narwal S. Kumar D. Sheoran S. Verma R. P. S. Gupta R. K. (2017). Hulless barley as a promising source to improve the nutritional quality of wheat products[J]. *J. Food Sci. Technol.* 54 2638–2644. 10.1007/s13197-017-2669-6 28928503 PMC5583093

[B47] Oh J. Y. Jee S. N. Nam Y. Lee H. Ryoo M. I. Kim K. D. (2007). Populations of fungi and bacteria associated with samples of stored rice in Korea.[J]. *Mycobiology* 35 36–38. 10.4489/MYCO.2007.35.1.036 24015066 PMC3763084

[B48] Palacios S. A. Del Canto A. Erazo J. Torres A. M. (2021). Fusarium cerealis causing Fusarium head blight of durum wheat and its associated mycotoxins. *Int. J. Food Microbiol.* 346:109161. 10.1016/j.ijfoodmicro.2021.109161 33773354

[B49] Patriarca A. (2016). Alternaria in food products[J]. *Curr. Opin. Food Sci.* 11 1–9. 10.1016/j.cofs.2016.08.007

[B50] Patriarca A. (2019). Fungi and mycotoxin problems in the apple industry. *Curr. Opin. Food Sci.* 29 42–47. 10.1016/j.cofs.2019.08.002

[B51] Podgórska-Kryszczuk I. Solarska E. Kordowska-Wiater M. (2022). Biological Control of Fusarium culmorum, Fusarium graminearum and Fusarium poae by Antagonistic Yeasts. *Pathogens* 11:86. 10.3390/pathogens11010086 35056034 PMC8777846

[B52] Rajeev B. Ravishankar V. R. Karim A. A. (2010). Mycotoxins in Food and Feed: present Status and Future Concerns[J]. *Comprehens. Rev. Food Sci. Food Saf.* 9 57–81. 10.1111/j.1541-4337.2009.00094.x 33467806

[B53] Rashim K. Virat A. Mahesh G. (2018). Nutritional, functional and textural properties of healthy snacks formulation from hulled and hull-less barley[J]. *J. Food Meas. Charact.* 12 1219–1228. 10.1007/s11694-018-9736-1

[B54] Romero F. Cazzato S. Walder F. Vogelgsang S. Bender S. F. van der Heijden M. G. A. (2022). Humidity and high temperature are important for predicting fungal disease outbreaks worldwide. *New Phytol.* 234 1553–1556. 10.1111/nph.17340 33713447

[B55] Sindt C. Besancenot J. P. Thibaudon M. (2016). Airborne Cladosporium fungal spores and climate change in France[J]. *Aerobiologia* 32 53–68. 10.1007/s10453-016-9422-x

[B56] Sun D. Qu J. Huang Y. Lu J. Yin L. (2021). Analysis of microbial community diversity of muscadine grape skins[J]. *Food Res. Internat.* 145 110417–110417. 10.1016/j.foodres.2021.110417 34112420

[B57] Susana A. Elena G. P. María M. A. Adela L. C. (2008). Ochratoxin A decontamination: A review[J]. *Food Control* 20 326–333. 10.1016/j.foodcont.2008.05.017

[B58] Tanaka M. A. S. Maeda J. A. Plazas I. H. A. Z. (2001). Microflora fúngica de sementes de milho em ambientes de armazenamento[J]. *Scientia Agricola* 58:501.

[B59] Tralamazza S. M. Bemvenuti R. H. Zorzete P. de Souza Garcia F. Corrêa B. (2012). The genus Cladosporium[J]. *Stud. Mycol.* 72 1–401. 10.3114/sim0003 22815589 PMC3390897

[B60] Tralamazza S. M. Bemvenuti R. H. Zorzete P. (2016). Fungal diversity and natural occurrence of deoxynivalenol and zearalenone in freshly harvested wheat grains from Brazil. *Food Chem.* 196 445–450. 10.1016/j.foodchem.2015.09.063 26593513

[B61] Wang L. Liu B. Jin J. Ma L. Dai X. Pan L. (2019). The Complex Essential Oils Highly Control the Toxigenic Fungal Microbiome and Major Mycotoxins During Storage of Maize[J]. *Front. Microb.* 10:1643. 10.3389/fmicb.2019.01643 31379790 PMC6646819

[B62] Wang Q. Garrity G. M. Tiedje J. M. Cole J. R. (2007). Naive Bayesian classifier for rapid assignment of rRNA sequences into the new bacterial taxonomy. *Appl. Env. Microbiol.* 73 5261–5267. 10.1128/AEM.00062-07 17586664 PMC1950982

[B63] Windes S. Bettenhausen H. M. Simaeys K. R. V. Clawson J. Fisk S. Heuberger A. L. (2020). Comprehensive Analysis of Different Contemporary Barley Genotypes Enhances and Expands the Scope of Barley Contributions to Beer Flavor[J]. *J. Am. Soc. Brew. Chem.* 79 281–305. 10.1080/03610470.2020.1843964

[B64] Woudenberg J. H. Groenewald J. Z. Binder M. Crous P. W. (2013). Alternaria redefined. *Stud. Mycol.* 75 171–212. 10.3114/sim0015 24014900 PMC3713888

[B65] Xie T. Shen S. Hao Y. Li W. Wang J. (2022). Comparative Analysis of Microbial Community Diversity and Dynamics on Diseased Tubers During Potato Storage in Different Regions of Qinghai China.[J]. *Front. Genet.* 13:818940–818940. 10.3389/FGENE.2022.818940 35273638 PMC8902257

[B66] Xu D. Xue M. Shen Z. Jia X. Hou X. Lai D. (2021). Phytotoxic Secondary Metabolites from Fungi. *Toxins* 13:261. 10.3390/toxins13040261 33917534 PMC8067579

[B67] Yang F. (2021). Prediction of the growth of fungal communities in different environments: a GomPertz model approach. *Earth Env. Sci.* 784:1. 10.1088/1755-1315/784/1/012046

[B68] Yao Y. Long M. (2020). The biological detoxification of deoxynivalenol: a review. *Food Chem. Toxicol.* 145:111649. 10.1016/j.fct.2020.111649 32745571

[B69] Ye J. Joseph S. D. Ji M. Nielsen S. Mitchell D. R. G. Donne S. (2017). Chemolithotrophic processes in the bacterial communities on the surface of mineral-enriched biochars[J]. *ISME J.* 11:1087. 10.1038/ismej.2016.187 28169988 PMC5437921

[B70] Youenou B. Favre-Bonté S. Bodilis J. Brothier E. Dubost A. Muller D. (2015). Comparative genomics of environmental and clinical *stenotrophomonas* maltophilia strains with different antibiotic resistance profiles[J]. *Gen. Biol. Evol.* 7 2484–2505. 10.1093/gbe/evv161 26276674 PMC4607518

[B71] Zhang Q. Li D. Zhang W. Jiang M. Chen X. H. Dong M. S. (2021a). Comparative analysis of the bacterial diversity of Chinese fermented sausages using high-throughput sequencing. *LWT* 2021:150. 10.1016/j.lwt.2021.111975

[B72] Zhang Q. Shi W. C. Zhou B. Du H. Y. Xi L. Q. Zou M. (2021b). Variable characteristics of microbial communities on the surface of sweet cherries under different storage conditions[J]. *Postharv. Biol. Technol.* 173:111408. 10.1016/J.POSTHARVBIO.2020.111408

[B73] Zhao H. Chu M. Huang Z. Yang X. Ran S. Hu B. (2017). Variations in oral microbiota associated with oral cancer[J]. *Scient. Rep.* 7:11773. 10.1038/s41598-017-11779-9 28924229 PMC5603520

[B74] Zhou Y. J. Li J. H. Ross Friedman C. Wang H. F. (2017). Variation of soil bacterial communities in a chronosequence of rubber tree (Hevea brasiliensis) plantations[J]. *Front. Plant Sci.* 8:849. 10.3389/fpls.2017.00849 28611794 PMC5447074

